# Growth Trajectories in Psychological Well-Being: A Clinical Trial for Older Adults with Macular Degeneration

**DOI:** 10.1093/geroni/igaf122.1742

**Published:** 2025-12-31

**Authors:** Piaopiao Cai, Timothy Powers, Silvia Sörensen

**Affiliations:** University of Rochester, Rochester, New York, United States; University of Rochester, Rochester, New York, United States; University of Rochester Warner School of Education and Human Development, Rochester, New York, United States

## Abstract

Age-related macular degeneration (AMD) affects over 20% of aging populations (Lim et al., 2012). AMD is a significant cause of visual impairment and severe vision loss (Mitchell et al., 2018), affecting around 37 million Americans aged 50 and above (Pelletier et al., 2016). Our study aims to understand growth trajectories of psychological well-being (autonomy, environmental mastery, personal growth, positive relationships, and self-acceptance), utilizing a five-wave clinical trial study (Sörensen et al., 2015), that randomized participants into two groups: Treatment (Preventive Problem-Solving Intervention (PREPSI, n = 90)); Social Contact Control (“Life and Health Review Intervention” (LIHRI, n = 90)). Mixed-model ANOVAs showed a significant decline in perceptions of autonomy, environmental mastery, purpose in life, and self-acceptance after five waves (in both Intention to Treat (ITT) and Completed Case (CC) analysis), but stability in positive relationships with others and a marginally significant quadratic effect for personal growth in the CC analysis. Treatment and control groups differed significantly in trajectories of environmental mastery and self-acceptance; PREPSI respondents had significantly higher scores on these two outcomes after wave 4, while those in the control group exhibited a marked decrease. The minimum and maximum turning points of these trajectories were identical in CC analysis. Additionally, the strong variation and significant cubic change in the trajectories of purpose in life for both groups indicate that the PREPSI did not significantly enhance the purpose in life trajectory. The steady decline in wellbeing among people with vision loss and underlying individual and contextual influences require further investigation to guide future intervention development.

